# Different ^212^Pb Generators and Its Radiation Safety Concerning ^220^Rn (Thoron) Emanation

**DOI:** 10.3390/toxics13060462

**Published:** 2025-05-30

**Authors:** Marc Pretze, Holger Hartmann, Charlotte Duchemin, Thierry Stora, Muhammad Inzamam, David Kästner, Edwin A. Sagastume, Michael K. Schultz, Jörg Kotzerke, Ralph A. Bundschuh, Robert Freudenberg

**Affiliations:** 1 Department of Nuclear Medicine, University Hospital Carl Gustav Carus, Technical University Dresden, Fetscherstr. 74, 01307 Dresden, Germanyjoerg.kotzerke@ukdd.de (J.K.); robert.freudenberg@ukdd.de (R.F.); 2 CERN, 1217 Meyrin, Switzerland; charlotte.duchemin@cern.ch (C.D.); thierry.stora@cern.ch (T.S.);; 3 Perspective Therapeutics, Coralville, IA 52241, USA; 4 Department of Radiology, The University of Iowa, Iowa City, IA 52240, USA; 5 Department of Chemistry, The University of Iowa, Iowa City, IA 52242, USA

**Keywords:** ^224^Ra, ^220^Rn, ^212^Pb, alpha detection, dosimetry, radiation safety, radiolabeling

## Abstract

(1) Background: As the demand for ^212^Pb for clinical theranostics rises, empirical studies that examine the radiation safety implications of different ^224^Ra sources are needed to facilitate discussions with local authorities for the translation of ^203/212^Pb theranostics routine clinical practice. (2) Methods: Environmental ^220^Rn (Thoron) emanation was detected by a RAD7 detector in the vicinity of respective ^212^Pb sources and additional alpha-dosimeters to detect ^220^Rn during generator elution, radiosynthesis, and quality control. Personnel gamma exposure was measured using whole-body and ring dosimeters. Generators included those based on wet-chemical-process- and emanation-based technology. (3) Results: During generator handling, varying levels of ^220^Rn were observed in the vicinity of generators. An additional monthly whole-body dose must be considered when handling different sources of ^212^Pb generators, and this depends upon local shielding and the handling approaches toward use of the technology. (4) Conclusions: ^224^Ra in any form (including radionuclide generators) should always be handled within a fume hood to keep potential contamination and exposure to personnel as low as reasonably achievable. Following standard practices of radiation safety, generators of ^212^Pb can be used safely for theranostic applications.

## 1. Introduction

^212^Pb (half-life *t*_1/2_ = 10.64 h; 0.57 MeV β^–^ emission; 100% intensity) is a promising radionuclide for targeted alpha-particle therapy [1] that decays to ^212^Bi (*t*_1/2_ = 60.6 min) and then emits one α-particle, either with a probability of 64% by decaying to ^212^Po (*t*_1/2_ = 0.3 µs) and ^208^Pb or with a probability of 36% by decaying to ^208^Tl (*t*_1/2_ = 3.1 min) and ^208^Pb [2,3]. Therefore, a ^212^Pb-labeled radiopharmaceutical, once accumulated in the tumor tissue, will deposit its highest dose in the form of the α-particle specifically in the tumor cells, with a lower probability of further α-decay occurring in healthy organs. Thus, ^212^Pb represents a more favorable choice for cancer α-therapy of patients who are naïve to (or who have progressed on) β–therapy, including patients with reduced renal function. Ongoing preclinical and clinical studies are investigating the potential of ^212^Pb-labeled peptides and antibodies (with administered radioactivity in the range of approximately 1–3 MBq/kg) [4] (bridging the gap between administered activities of ^177^Lu and ^225^Ac). Recently, the true matched pair ^203/212^Pb has come into focus through several first in-human theranostic applications [5,6]. While ^203^Pb (*t*_1/2_ = 51.9 h; 279 keV gamma ray; 81% intensity) represents an ideal elementally matched imaging surrogate, ^212^Pb itself can also be applied for SPECT imaging [7,8]. This true matched pair has the potential to overcome the differential pharmacokinetic/pharmacological properties observed between diagnostic and therapeutic radiotracers with unmatched radionuclide pairs like ^68^Ga/^177^Lu [9]. The radiopharmaceutical VMT-α-NET comprises a lead-specific chelator (PSC) conjugated to a [Tyr^3^,Thr^8^]octreotide backbone via a polyethylene glycol linker (PEG_2_). Thus, this radiopharmaceutical is a somatostatin subtype 2 (SST2) receptor-targeting peptide that can be used for the imaging and treatment of neuroendocrine tumors (NETs) that exhibits rapid tumor accumulation, high tumor retention, and rapid renal excretion [10]. It carries the chelator PSC [10], which forms highly stable complexes with ^203/212^Pb and, in contrast to less stable 1,4,7,10-tetraazacyclododecane-*N*,*N*’,*N*”,*N*’”-tetraacetic acid (DOTA) complexes, remains intact even after β^–^ conversion to ^212^Bi [11]. Another known chelator for ^203/212^Pb is DOTAM (also known as TCMC), which was already used in a clinical dose escalation trail with ^212^Pb-DOTAM-TATE [12], for which instability of the ^212^Pb radionuclide progeny ^212^Bi has been reported [13].

**Figure 1:**
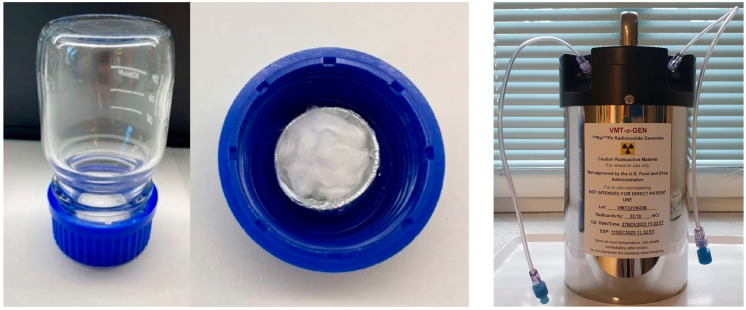
Prototype ^224^Ra/^212^Pb generator from CERN (duran-flask-emanation-based) and the wet-chemical-based ^224^Ra/^212^Pb generator (VMT-α-GEN) from Perspective Therapeutics.

Access to ^212^Pb remains limited, and the available generators are in different experimental [14,15] or preclinical stages [10]. For radiation safety and risk assessment, two ^212^Pb generators were obtained. Alpha and gamma dosimetry were performed during generator elution and radiosynthesis (Figure 1). Key parameters in this context included observed emanation of ^220^Rn (Thoron) (*t*_1/2_ = 55.6 s) during generator elution and monitoring of the 2.6 MeV gamma radiation from ^208^Tl during radiosynthesis, which were rated as critical for radiation exposure of personnel. Importantly, radiation safety measures can be easily taken to minimize the exposure to personnel for both of these parameters.

**Figure 2:**
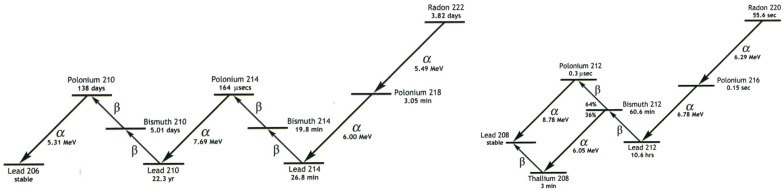
Decay schemes of radon ^222^Rn (**left**) and ^220^Rn (**right**), with respective decay energies for detection by the RAD7 detector.

For the detection and measurement of ^220^Rn, an electronic radon detector (RAD7, DURRIDGE, Billerica, MA, USA) was used. The interworking of the RAD7 consisted of a solid-state, ion-implanted, planar, silicon-alpha detector housed within a 0.7 L hemisphere. The RAD7 specifically detects the alpha energies of ^218^Po (*t*_1/2_ = 3.05 min, 6.00 MeV, channel A) and ^216^Po (*t*_1/2_ = 0.15 s, 6.78 MeV, channel B) as decay products from ^222^Rn (*t*_1/2_ = 3.82 d) and ^220^Rn, respectively, which can be used to quantify the concentration of each of these Rn isotopes (Figure 2). For our measurements relevant here, due to the short half-life of ^216^Po, the response of the RAD7 to ^220^Rn is virtually instantaneous, while the response to ^222^Rn will take 15 min for the count rate to reach equilibrium. The upper limit of the RAD7 is 1 MBq/m^3^. The original tubing from the manufacturer was used, as the detector was calibrated to the length of the tubing.

In this work, the results of ^220^Rn measurements and dosimetry of personnel over two years of generator elution, radiosynthesis, and quality control are presented. Thus, we intend to provide an informed overview of the safe operations of the two generator systems and information from our experience that can be useful for planning the handling ^212^Pb sources in laboratories with similar fume hoods. Our radiation protection required handling under these conditions. It is expected that this work will help other departments to consider radiation safety measures to be implemented that have a scientific basis for discussion internally and with their local authorities.

## 2. Materials and Methods

All reagents and solvents were purchased from commercial suppliers at the highest purity and used without further purification. VMT-α-NET (PSC-PEG_2_-TOC) and ^224^Ra/^212^Pb generator (VMT-α-GEN) were obtained from Perspective Therapeutics Inc. (Coralville, IA, USA). Another ^224^Ra/^212^Pb generator (CERN generator) was obtained from MEDICIS (CERN, CH-1211 Geneve 23, Switzerland). Custom-made Pb resin cartridges were filled with 50 mg of powder (PB-B10-F, Triskem, Bruz, France). The RAD7 electronic radon detector was obtained from DURRIDGE (Billerica, MA 01821, USA). Radiochemical purity (RCP) was monitored by TLC on iTLC-SG plates (Agilent, Santa Clara, CA, USA). Measurement of the radionuclide purity (RNP) and evaluation of the radio-TLC was performed with a thin-layer scanner (MiniScanPRO+, Eckert&Ziegler Eurotope GmbH, Berlin, Germany) equipped with a Model 43-2 alpha detector ZnS(Ag) scintillator (Ludlum Measurements, Sweetwater, TX, USA) and a built-in multi-channel analyzer (MCA) for gamma spectroscopy. Radio-HPLC was performed on a Shimadzu HPLC system (Thermo Scientific, Dreieich, Germany) equipped with a reverse-phase column (Analytical: Merck Chromolith HighResolution RP-18e; 150 × 4.6 mm plus a 5 × 4.6 mm guard column, Darmstadt, Germany) and a UV diode array detector (220 nm). The solvent system used was a gradient of acetonitrile–water (containing 0.05% TFA) (0–13 min: 0–60% MeCN) at a flow rate of 1.6 mL/min, unless otherwise stated. The pH was measured using a reflectance photometer (QUANTOFIX Relax, Macherey-Nagel GmbH & Co. KG, Düren, Germany). For personal dosimetry, whole-body dosimeters (type: LPS-OSL-GD 01, LPS, Berlin, Germany) and ring dosimeters (type: LPS-TLD-TD 08, Thermo Fisher Scientific, Berlin, Germany) were used.

### Radiochemistry

Radiolabeling of the tumor ligands conjugated either with the chelators TCMC (DOTAM) or PSC was performed according to reported standard protocols for these chelators [11]. Briefly, 50 µg of precursor VMT-α-NET (PSC-TOC derivative, M = 1578.7 g/mol)) or 62 µg of PSV-402 (TCMC-PSMA derivative, M = 1675.7 g/mol) (1 µg/µL in H_2_O_suprapure_) was added to a 10 mL reaction vial together with 100 µL of EtOH_absolute_, 290 µL of 1 M NaAc/AcOH buffer (pH 4, 99.99% trace metal), and 2 mg of sodium ascorbate (Ph.Eur.).

In general, the elution of the generators was performed within a safety fume hood in order to avoid the release of ^220^Rn into the laboratory. During elution, the RAD7 was in sniffing mode with ^220^Rn detection activated. The sniff tube was attached in the vicinity of the respective generator outlet. During elution, the personnel wore additional FFP2 masks and arm cuffs in order to prevent contamination by skin adsorption of ^212^Pb from ^220^Rn.

Elution of VMT-α-GEN was performed with 4 mL of 2 M HCl_suprapure_, followed by 4 mL of air and an additional 1 mL of H_2_O_suprapure_ in order to wet the generator and avoid further ^220^Rn release from the generator.

Elution of CERN generator was performed by rapidly changing the glass vial with another one and letting the glass vial containing the ^220^Rn stay on top for about 10 min in order to let the ^220^Rn decay. Subsequently, the glass vial was rinsed 1–4 times with 1 mL 0.1 M HCl_suprapure_.

The collected generator eluates of ^212^Pb in 3–5 mL 0.1–1.6 M HCl_suprapure_ were trapped on a custom-made Pb resin cartridge (50 mg PB-B10-F, Triskem, Bruz, France) preconditioned with 1 mL of 2 M HCl_suprapure_. The captured activity was rinsed with 1 mL of 2 M HCl_suprapure_. The activity was eluted with 2 mL of NaAc/AcOH buffer (pH 6, 99.99% trace metal) directly into the reaction vial. The solution was heated at 95 °C for 30–60 min. The reaction solution was then diluted with 4 mL of 0.9% NaCl solution and cooled.

**Figure 3:**
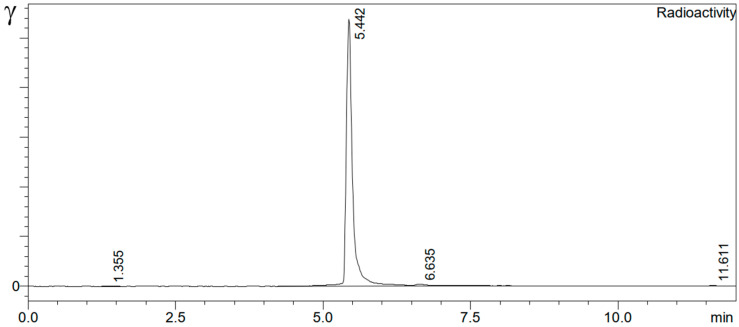
Representative radio-chromatogram of ^212^Pb-PSV-402 of the reaction solution indicating quantitative complexation of ^212^Pb and ^212^Bi.

Finally, the product was purified by using a C18 Plus light cartridge (WAT023501, Waters, Eschborn, Germany) preconditioned with 1 mL of EtOH and 3 mL of H_2_O (wet condition). The cooled and diluted product solution (4 mL of 0.9% NaCl) was slowly passed through the C18 cartridge. The C18 cartridge containing the product was rinsed with 2 mL of 0.9% NaCl solution and was directly eluted with 1 mL of 50% EtOH for injection directly through a vented sterile filter (0.22 µm, SLGVV255 F, Millex-GV, Merck-Millipore) into a product vial. Finally, the product was diluted with 7 mL of 0.9% NaCl solution. The radiochemical yield for ^212^Pb-VMT-α-NET and ^212^Pb-PSV-402 were >95%, and the radiochemical purities were >99% (Figure 3), respectively.

## 3. Results

**Figure 4:**
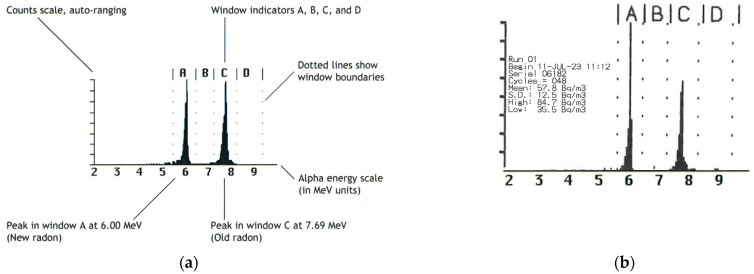
(**a**) Example of alpha energy spectrum of environmental measurement with RAD7 detector. ^218^Po has an energy of 6.00 MeV, and ^214^Po has an energy of 7.69 MeV; (**b**) occurrence of natural ^222^Rn within the fume hood in the vicinity of the ^212^Pb generator.

Prior to the generator handling, a two-day background measurement within the fume hood in the vicinity of the respective generator was performed and compared with an example spectrum without a generator (Figure 4a), which resulted exclusively in the detection of natural ^222^Rn (57.8 ± 12.5 Bq/m^3^) (Figure 4b).

**Figure 5:**
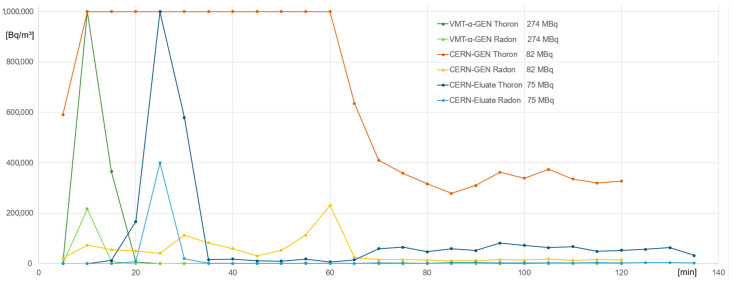
Concentration of ^220^Rn (Thoron) and ^222^Rn (Radon) in Bq/m^3^ within the fume hood measured by the RAD7 prior to and during generator elution (0–30 min), radiosynthesis (30–60 min), and subsequent quality control (60–135 min).

**Figure 6:**
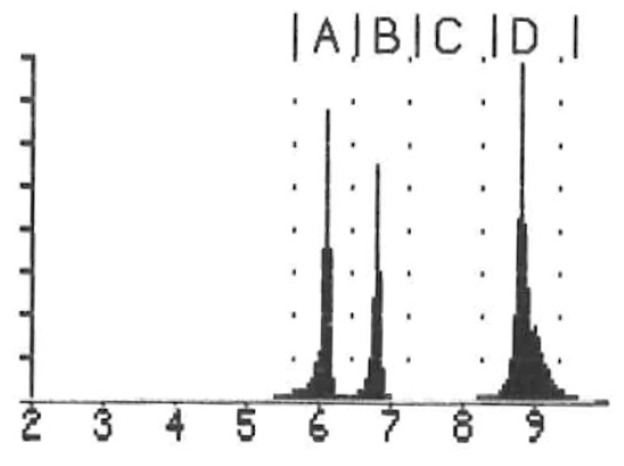
Representative spectrum of ^220^Rn measurement during generator elution of VMT-α-GEN showing ^220^Rn in equilibrium. ^212^Bi has an energy of 6.05 MeV, ^216^Po has an energy of 6.78 MeV, and ^212^Po has an energy of 8.78 MeV.

No ^220^Rn emanation was detected when the generators were stored within the fume hood (wet storage for VMT-α-GEN and dry for CERN generator) and without use. The concentrations of ^220^Rn and ^222^Rn in Bq/m^3^ over the time of handling for both generators are displayed in Figure 5. The measurements were stopped when an equilibrium was reached or after the end of a whole synthesis (120–135 min), including C18 purification and quality control by thin-layer chromatography (TLC). ^220^Rn emanation was measured during generator elution and radiolabeling. Measurable ^220^Rn release was observed only during generator elution (Figure 6), and as soon as the eluate was trapped on the Pb resin, the ^220^Rn signal from the air decreased significantly. For example, the elution of 493 MBq ^212^Pb from VMT-α-GEN resulted in a release of ^220^Rn with >1 MBq/m^3^ for 5 min, which then decreased to 0.4 MBq/m^3^ after 10 min and <0.1 MBq/m^3^ after 15 min.

Elution of ^212^Pb from CERN generator (~700 MBq EOB; ~600 MBq at shipping time; ~500 MBq at reception time) was carried out by rinsing the glass bottle with 1–4 mL of 0.1 M HCl. This resulted in a higher release of ^220^Rn with >1 MBq/m^3^ for 55 min, reaching a lower plateau of <0.4 MBq/m^3^ for the rest of the measurement. The recovery rate of the rinsing process is shown in Table 1. Therefore, at least 3 mL of 0.1 M HCl had to be used for effective activity recovery from the glass bottle. Lower ^220^Rn release was observed when switching from rinsing the glass bottle with HCl to completely dissolving the ^224^Ra/^212^Pb from the glass wool in 1 M HCl, passing the solution over the Pb resin (^212^Pb trapping), and collecting the ^224^Ra-containing eluate back into the residual vial. Here, ^220^Rn with 0.6–0.66 MBq/m^3^ in the first 10 min was measured, which dropped below 0.08 MBq/m^3^ after 15 min and further. Therefore, a significant amount of ^220^Rn could be prevented from escaping into the air. Here, the recovery rate of ^212^Pb was at least 66 ± 2.5% (n = 3), and therefore, the procedure was superior to the glass bottle method.

### 3.1. Direct Measurement of Radiation Exposure

Radiation exposure of a VMT-α-GEN generator (360 MBq) was measured with standard well-known dosimeters under different conditions. The dose rate at a distance of 1 m and on the surface was 18.2 µSv/hr and 3.5 mSv/hr, respectively. A dose rate of 10 µSv/h was measured at a distance of 1 m for an unshielded vial with 32 MBq. Attenuation factors of 1.5, 2.2, and 8.2 were determined for lead absorbers with thicknesses of 0.5 cm, 1 cm, and 5 cm. By means of a test irradiation on an Alderson phantom, various electronic personal dosimeters (EPDs) were examined with regard to their suitability and compared with the measured values of two official personal dosimeters. The mean value of the EPDs used was (0.72 ± 0.15) µSv, thus showing good agreement with the values of the official dosimeters (0.7 mSv and 0.8 mSv).

### 3.2. Radiation Exporsure to Medical Staff

Radiation exposure of personnel in our department is routinely monitored by measuring whole-body (film dosimeter type LPS-OSL-GD 01) and partial body (finger ring OSL dosimeter) doses. Looking at the values for nearly two years, it can be found that there were slightly higher effective and finger doses in the months when the ^212^Pb generators were handled (Table 2, one person). It should be noted that our experience has assisted us in establishing procedures that minimize whole-body and finger ring exposures. On average, the starting activity was ~600 MBq, and the generators were eluted every 1–2 days for up to four weeks (first week for patient use and the following three weeks for experimental radiochemistry). The mean whole-body and finger doses accumulated during clinical routine production of radiotracers were (0.133 ± 0.047) mSv and (6.583 ± 5.649) mSv per month, respectively. The mean effective and finger doses accumulated in the months of generator handling were (0.250 ± 0.126) mSv and (8.083 ± 3.989) mSv per month, respectively. Therefore, an additional monthly effective dose of ~0.12 mSv can be expected when handling these types of generators. This would result in an additional dose of 1.44 mSv per year for one person if a fresh ^224^Ra/^212^Pb generator were to be handled every month.

An attempt was made to assess potential ^220^Rn levels that might be observed in the breathing zone of operators for the case where a generator was located outside of an appropriate fume hood hot-cell working environment. In this case, generators were eluted, and operators wore FFP2-masks during the elution of approximately 600 MBq starting activity through the Pb resin trapping step. Subsequently, potential activity trapped by the masks was assayed by the alpha-detection mode of a CoMo-170 device (NUVIA Instruments, Dresden, Germany). Although this approach is a crude measurement of radioactivity, no signal above the 50 IPS (warning signal) was detected (background of ~10 IPS). These results demonstrate the importance of operation of generator technologies in an appropriate fume hood that is shielded according to ALARA principles.

### 3.3. ^222^Rn Exposure

As a higher ^222^Rn signal was detected during generator elution, an additional ^222^Rn dosimetry to the gamma dosimetry by three ^222^Rn exposimeters (type: B97, ALTRAC, Berlin, Germany) was performed during the experiments to rule out ^222^Ra emanation, since the generators are known to be absolutely free of any ^226^Ra due to the production process of ^224^Ra [16]. One was positioned inside the fume hood within the vicinity of the ^212^Pb-generator, one was positioned outside of the fume hood, and one was attached to the lab coat of the personnel. Unfortunately, the exposimeters were restricted to detect ^222^Rn only. A slightly higher ^222^Rn level was measured within the fume hood in the vicinity of the ^212^Pb generator, which was expected due to a higher measured activity of ^222^Rn with the RAD7 during elution (see Table 1). As the differences between the measured values were very small, this could have naturally occurred, since there was only one dosimeter used at the respective measuring points. However, the measured values of the dosimeters were 443 kBq h/m^3^ in the vicinity of the ^212^Pb generator, 364 kBq h/m^3^ outside the fume hood, and 111 kBq h/m^3^ at the lab coat. Therefore, the detection of ^222^Rn during the detection of high concentration of ^220^Rn might have been due to measurement issues of the RAD7 itself and not because of some small impurities of ^226^Ra within the ^224^Ra.

## 4. Discussion

As discussed in the literature, ^224^Ra itself could be used for alpha therapy (DaRT) [17], and therefore, the emission of ^220^Rn in vivo is of another level of importance. Recently, the first preclinical results of ^220^Rn diffusion in tissue were obtained [18]. Based on this, calculations were performed in order to determine the dosimetry in vivo [19]. Parallel to the brachytherapeutic concept of using alpha-particles for therapy, the approach presented here is based on the radiolabeling of tumor-specific ligands with ^212^Pb. The detection system employed (i.e., RAD7) is the only transportable system commercially available to our knowledge for the measurement of the radionuclide relevant to our application (i.e., ^220^Rn), as other personal alpha detectors were only able to detect naturally occurring ^222^Rn and were therefore unsuitable for this purpose. Other departments like PTB are able to measure probes of ^220/222^Rn [20].

*Elution of CERN generator and workup of ^224^Ra solution*

In our study, the elution efficiency of ^212^Pb from the glass bottle was ~30%, in contrast to the 80–85% reported in the literature [14]. The radiolabeling with the eluate from CERN generator for, e.g., DOTA peptide resulted in a ~50% radiochemical yield (RCY) when using the same precursor amounts. For a higher radiolabeling yield, the amount of precursor had to be increased to 50–62 µg vs. 20 µg. Therefore, and because of a high release of ^220^Rn during rinsing of the bottle, it cannot be recommended to use ^212^Pb in this form with our present infrastructure. In order to prevent ^220^Rn release, the glass wool containing the ^224^Ra was placed in 1–2 mL of 1 M HCl in order to dissolve the ^224^Ra and ^220^Rn. This resulted in an order of magnitude lower release of ^220^Rn by the wet method. However, from that experience onwards, further ^224^Ra derived from CERN generator was already provided in solution (1 M HCl), preventing higher ^220^Rn release by dissolving the glass wool containing ^224^Ra in the laboratory.

*Elution of VMT-a-GEN*

The elution yield of the ^224^Ra/^212^Pb generator varied between 59–80% of the total loaded ^224^Ra activity, depending on days between consecutive generator elution [21]. The recovery rate with 66% for ^212^Pb from the ^224^Ra solution (CERN generator) was in-between the recovery rate of VMT-α-GEN, whereas the recovery rate of ^212^Pb from CERN generator was the lowest observed at ~30%. Notably, the ^220^Rn release of VMT-α-GEN was the lowest of the three different types of ^212^Pb separation examined. From this work, VMT-α-GEN is suggested as the best and safest choice for handling ^212^Pb.

*Pb resin trapping*

**Figure 7:**
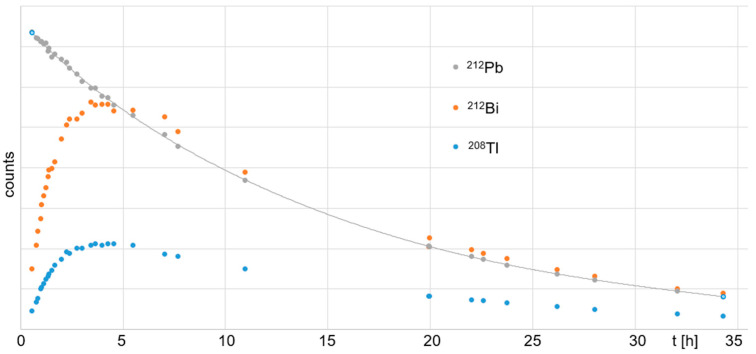
Curve of decay for pure ^212^Pb eluate from Pb resin elution and ingrowth of daughter nuclides ^212^Bi and ^208^Tl for 35 h.

**Figure 8:**
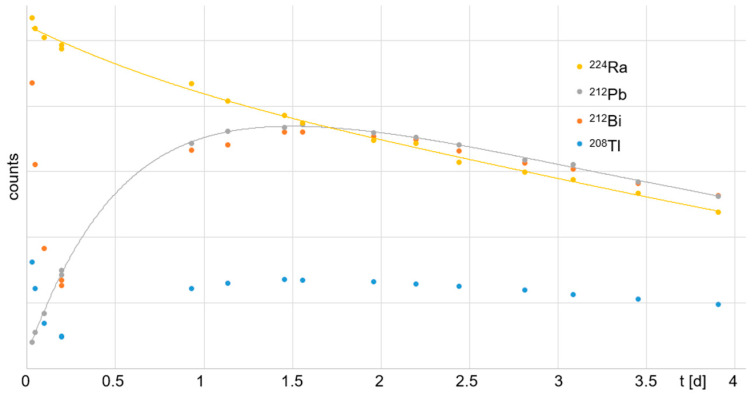
Curve of decay for residual ^224^Ra separated from ^212^Pb by resin and ingrowth of daughter nuclides within four days. The initial activity of the daughter nuclides ^212^Bi and ^208^Tl decreased significantly within the first 20 min because they are additional radionuclides obtained from the equilibrium state prior to elution and separation from ^212^Pb.

All isotopes of Pb were trapped by the Pb resin exclusively. The ^212^Pb eluate measured ~60% of the starting activity, where all nuclides were in equilibrium. All other nuclides, ^224^Ra, ^212^Bi, and ^208^Tl, were found in the waste elution, which measured ~40% of the starting activity. Therefore, the waste elution could be recycled and reused for further experiments within the following days. In Figure 7, the ingrowth of daughter nuclides over time for the Pb-resin-eluted ^212^Pb solution is shown, as is the radioactive decay of the initial ^212^Pb with a half-life of (10.7 ± 0.2) h. The residual eluate that was separated from the ^212^Pb by the Pb resin contained ^224^Ra, ^212^Bi, and ^208^Tl (Figure 8). ^212^Bi and ^208^Tl decayed initially, with their respective half-lives of *t*_1/2_ = 60.6 min and 3.1 min. At ~0.2 d (~4.8 h), their decay reached equilibrium with the newly formed ^212^Pb, which resulted from the ^224^Ra decay. ^212^Pb showed a typical ingrowth to equilibrium with its mother nuclide ^224^Ra over time. However, the ^212^Pb eluate showed a typical ingrowth of the daughter nuclides ^212^Bi and ^208^Tl up to equilibrium with their mother nuclide ^212^Pb (~4 h after Pb resin purification). This provides a method for the straightforward handling of waste by fast decay in storage.

The measured additional monthly effective dose of ~0.12 mSv would result in a calculated additional dose of 1.44 mSv per year for the operator if a fresh ^224^Ra/^212^Pb generator were to be handled every month. In comparison, a monthly effective dose of ~0.54 mSv (mean value for three MTAs over two years exclusively working in our PET facility) can be found for handling ^11^C/^18^F/^68^Ga radiopharmaceuticals for patient application purposes. Personnel dose is restricted to 20 mSv per year; therefore, a dose of 1.44 mSv is 7% of the annual allowed dose, which indicates a very low additional risk. However, several methods such as varying the personnel or improvements to the time, shielding, and distance parameters are under consideration and will result in an even lower additional dose per year for one person.

The daughter nuclide ^208^Tl from ^212^Pb has a high gamma energy of 2.6 MeV [22]. It is assumed that most of the gamma radiation is not detected by the whole-body dosimeter. However, the gamma radiation may trigger “Bremsstrahlung” at the walls of the laboratory, which might then be detected by the dosimeter and would thus have a higher impact on the whole-body dose. Nevertheless, the generator itself was shielded by an additional 10 cm lead wall to reduce the breakthrough of the gamma radiation of ^208^Tl. In addition, VMT-α-GEN itself has a more effective and broader shielding (weight 35 kg) as compared to common ^188^Re or ^99m^Tc generators.

## 5. Conclusions

In conclusion, ^212^Pb generators can be safely eluted for the preparation of radiopharmaceuticals when operated within a closed fume hood or glovebox with sufficient shielding according to standard radiation safety ALARA principles and additional measures upon investigation of the workstation analysis driven with the Health and Safety and Environmental Protection (HSE) units. Simple measures in this context can be employed to provide a safe environment for operators. Once the ^212^Pb is trapped onto a Pb resin column, the activity can be handled outside the fume hood with appropriate consideration of the ingrowth of radionuclide progeny and, in particular, ^208^Tl (E_γ_ = 2.6 MeV). Appropriate shielding and hot-cell containment can be used to operate a generator system and mitigate unnecessary risk of the gamma rays from ^208^Tl and exposure to ^220^Rn during generator use. Under the conditions we provided in this study, an additional 0.1–0.2 mSv whole-body dose and a 1–2 mSv finger dose were accumulated within one month by eluting the generators and performing the radiosynthesis. Time, shielding, and distance fundamentals can easily be envisioned to further minimize the potential for exposure. Based on these findings, it can be concluded that the handling of the generators is safe under standard ALARA-principle-based planning of operation. In this study, the wet-chemical design of the generator (VMT-α-GEN) from Perspective Therapeutics demonstrated superior safety characteristics.

## Figures and Tables

**Table 1:** Recovery rate of ^212^Pb activity from glass bottle for rinsing steps (n = 4).

Volume of 0.1 M HCl	Mean Value of Recovered Activity
1 mL	15.8 ± 2.7%
2 mL	26.3 ± 2.4%
3 mL	30.0 ± 2.3%
4 mL	31.6 ± 2.7%

**Table 2:** Monthly accumulated effective doses (“body”) and finger doses in mSv over 2 years. Dates of arrival (**bold**) of ^224^Ra/^212^Pb generators were 16 March, 16 August, and 1 December 2023 and 6 May 26 July and 13 November 2024.

Dose	23 January	23 February	**23 March**	23 April	23 May	23 June	23 July	**23 August**	23 September	23 October	23 November	23 December
Body	0.1	0.1	**0.3**	0.1	0.1	0.1	0.1	**0.2**	0.2	0.1	0.2	**0.2**
Left *	4	5	**12**	1	7	3	3	**7**	11	2	8	**7**
Right *	8	6	**17**	2	11	3	2	**7**	6	2	6	**4**
Dose	24 January	24 February	24 March	24 April	**24 May**	24 June	**24 July**	24 August	24 September	24 October	**24 November**	24 December
Body	0.1	0.1	0.2	0.1	**0.1**	0.2	**0.5**	0.2	0.2	0.1	**0.2**	0.1
Left *	14	13	11	2	**14**	6	**8**	22	5	2	**5**	3
Right *	25	13	8	2	**6**	13	**7**	3	2	2	**3**	1

* Left and right refer to the dose values of the ring dosimeters worn at the left and right hand.

## Data Availability

Additional data can be requested by the corresponding author.
